# Determinants Associated With Pesticide Exposure in Patients With Head and Neck Cancer: Protocol for a Systematic Review

**DOI:** 10.2196/81312

**Published:** 2026-01-22

**Authors:** Gustavo Adolfo Sánchez-Ramírez, Manuel López Cabanillas Lomelí, Blanca Edelia González-Martínez, Ana Elisa Castro-Sánchez, Myriam Angélica De la Garza-Ramos, Guillermo Cano-Verdugo

**Affiliations:** 1 Universidad Autónoma de Nuevo León, Facultad de Odontología, Monterrey, Nuevo León, Mexico; 2 Universidad Autónoma de Nuevo León, Facultad de Salud Pública y Nutrición, Calle Dr. Eduardo Aguirre Pequeño No. 905, Col. Mitras Centro, CP.Monterrey, Nuevo León, 64460, México, 52 81 8329 4000 ext 3046

**Keywords:** occupational exposure, environmental risk factors, agrochemicals, rural health, cancer prevention, vulnerable populations

## Abstract

**Background:**

Currently, head and neck cancer (HNC) associated with pesticide exposure represents a global public health concern. However, there is no consensus regarding the specific determinants involved in this association. Moreover, there is a lack of scientific evidence to support the development of systematic reviews on this topic.

**Objective:**

This study aims to synthesize the methodology for conducting a systematic review to explore the current scientific evidence on the determinants of HNC associated with pesticides.

**Methods:**

The review will follow the PRISMA-P (Preferred Reporting Items for Systematic Review and Meta-Analysis Protocols) guidelines to ensure methodological rigor. The protocol includes detailed steps for constructing a robust search strategy using relevant databases such as PubMed, Embase, Scopus, Web of Science, CINAHL, LILACS, and AGRICOLA. Keywords and Medical Subject Headings terms related to “pesticides,” “exposure,” “head and neck neoplasms,” and related concepts will be used to capture the most relevant studies. Eligibility criteria will be clearly defined, including study design (eg, cohort, case-control, and cross-sectional), population characteristics, exposure assessment, and cancer outcomes. Studies published in any language that involve human participants will be included. The studies will be screened in 2 phases: first by title and abstract and then by full-text review. Two independent reviewers will assess the quality of each study and extract key data, such as exposure levels, cancer subtypes, and effect sizes. Any disagreements will be resolved through discussion or by a third reviewer.

**Results:**

This systematic review was initiated in February 2025 after protocol registration. The literature search and review process is ongoing at the time of submission of this paper. Data extraction and quality assessment have not yet been completed. Final results of the review, including a synthesis of determinants associated with pesticide exposure in patients with HNC, are expected to be completed and submitted for publication in June 2026.

**Conclusions:**

The necessary steps to conduct a systematic review must be concise and publicly available to ensure replicability within the scientific community.

## Introduction

Head and neck cancer (HNC) comprises a heterogeneous group of malignancies arising from the upper aerodigestive tract and represents a significant global public health concern, with more than 930,000 new cases and over 460,000 deaths reported worldwide in 2020 [1]. Within this group, oral cancer is one of the most common subtypes, accounting for nearly 377,000 cases annually. The increasing incidence of HNC and its subtypes, particularly in low- and middle-income countries, underscores the urgent need to understand the role of environmental and occupational determinants in their development [2].

According to the World Health Organization’s (WHO) Commission on Social Determinants of Health framework, health inequities arise from the unequal distribution of structural and intermediary determinants within societies. Structural determinants—such as socioeconomic status, education, occupation, and governance—shape the conditions of daily life, whereas intermediary determinants include material circumstances, psychosocial factors, and biological pathways through which exposures affect health outcomes. In the context of HNC, these determinants intersect with environmental and occupational exposures, such as pesticides, to modulate vulnerability and disease risk, particularly among rural and agricultural populations. Health outcomes are shaped by a complex interaction of biological, behavioral, social, and environmental determinants that influence individual and population well-being [3,4]. Among these, environmental exposures, especially to toxic or carcinogenic compounds, are increasingly recognized as major contributors to cancer etiology [5].

Pesticides, widely used in agricultural activities and vector-control programs in public health, represent one of the most relevant environmental exposures due to their potential mutagenic and carcinogenic effects. Chronic exposure to certain organophosphates, organochlorines, and carbamates has been associated with an increased risk of several malignancies, including leukemia, prostate cancer, and breast cancer. However, their specific contribution to oral carcinogenesis remains less explored. Mechanistically, pesticide exposure may promote HNC development through oxidative stress, DNA damage, epigenetic alterations, and chronic inflammatory pathways. Lipophilic compounds can accumulate in mucosal tissues, inducing cytotoxic and genotoxic effects that contribute to malignant transformation [6,7].

Despite emerging evidence suggesting a link between pesticide exposure and HNC risk, findings remain fragmented and inconsistent across studies, often due to methodological heterogeneity and limited population-based analyses. Therefore, this document presents a protocol for a systematic review aiming to synthesize the existing scientific evidence on the determinants associated with pesticide exposure in patients with HNC. This review seeks to clarify the potential causal mechanisms and to guide preventive strategies in public and occupational health contexts.

## Methods

### Protocol Registration

The review will follow the PRISMA-P (Preferred Reporting Items for Systematic Review and Meta-Analysis Protocols) [8] guidelines (Checklist 1) and is registered in PROSPERO (ID: CRD42024618939). This protocol was registered on December 6, 2024, and has 2 current versions (1 and 1.1). Through this protocol, we established the following focus question: What are the determinants associated with pesticide exposure in patients with HNC?

### Eligibility Criteria

Eligibility criteria will be based on the PECOS (population, exposure, comparator, outcome, and study) framework [9], considering the population as individuals who will have a confirmed diagnosis of HNC. Populations diagnosed exclusively with cancers other than HNC will be excluded. The primary clinical condition under investigation will be head and neck malignancies.

Exposure will be defined as populations with direct or indirect exposure to pesticides, regardless of the type or context of use. Studies where no form of pesticide exposure will be identified will not be considered. This decision aligns with previously reported challenges in evaluating low-level or undocumented exposure risks. Comparators will be considered as an unexposed or low-exposure population. Studies without a comparator will be included via consensus and only if they provide sufficient data to support synthesis.

Outcome will be HNC and will include demographic and exposure-related variables, such as age, sex, geographic location, ethnic background, occupational history, and substance use. Studies that will focus on nonrelevant variables, such as marital status, religious affiliation, number of offspring, sexual orientation, or personal hobbies, will be excluded. These demographic and behavioral variables will be considered essential for understanding the context of exposure and risk, consistent with frameworks for environmental epidemiology and cancer risk profiling.

Studies published within the last 20 years will be included to ensure the relevance and currency of the scientific evidence. This time frame is considered sufficient to capture the most significant developments in the field while excluding potentially outdated data that may no longer reflect current practices, technologies, or knowledge. Language restriction will not be considered, as we will work with a translation workflow if required. This will allow for a broad temporal scope and the inclusion of both historical and recent data.

Study design will include original research studies, such as cohort, case-control, and cross-sectional. Literature such as review articles, news media pieces, and academic theses will be excluded. The selection will be limited to sources that will offer original data and contribute to a robust and credible evidence base, as recommended in systematic review methodology standards.

### Search Strategy and Databases

A structured and reproducible search strategy will be developed and implemented across MEDLINE (via PubMed or Ovid), Embase, Scopus, Web of Science, CINAHL, LILACS, and AGRICOLA to identify relevant literature assessing the determinants in patients with HNC associated with pesticides, with a particular focus on cancers of the oral cavity. The search terms will be combined using Boolean operators to maximize sensitivity. It is anticipated that the search will cover literature from the 2000s to the present. The full search strategy and syntax are detailed in Table 1. Gray literature strategy will not be considered as consensus between authors.

**Table 1.** Proposed search strategy per database.

Database	Search strategy
PubMed	(“Head and Neck Neoplasms”[Mesh] OR “Oral Cancer”[tiab] OR “Laryngeal Cancer”[tiab] OR “Pharyngeal Cancer”[tiab] OR “Mouth Neoplasms”[tiab]) AND (“Pesticides”[Mesh] OR pesticide*[tiab] OR herbicide*[tiab] OR insecticide*[tiab] OR fungicide*[tiab] OR agrochemical*[tiab]) AND (“Environmental Exposure”[Mesh] OR “Occupational Exposure”[Mesh] OR “Agricultural Workers’ Diseases”[Mesh] OR determinant*[tiab] OR “environmental determinant*"[tiab])
Embase	('head and neck tumor’/exp OR ’oral cancer’/exp OR ’larynx cancer’/exp OR ’pharynx cancer’/exp) AND ('pesticide’/exp OR ’herbicide’/exp OR ’insecticide’/exp OR agrochemical*:ti,ab) AND ('environmental exposure’/exp OR ’occupational exposure’/exp OR determinant*:ti,ab)
Scopus	(TITLE-ABS-KEY(“HNC” OR “oral cancer” OR “pharyngeal cancer” OR “laryngeal cancer”)) AND (TITLE-ABS-KEY(pesticide* OR herbicide* OR insecticide* OR fungicide* OR agrochemical*)) AND (TITLE-ABS KEY(“environmental determinant*” OR “occupational exposure” OR “environmental exposure” OR determinant*))
Web of Science	TS=(“HNC” OR “oral cancer” OR “pharyngeal cancer” OR “laryngeal cancer”) AND TS=(pesticide* OR herbicide* OR insecticide* OR fungicide* OR agrochemical*) AND TS=(“environmental determinant*” OR “occupational exposure” OR “environmental exposure” OR determinant*)
CINAHL	(“Head and Neck Neoplasms” OR “Oral Cancer” OR “Pharyngeal Cancer” OR “Laryngeal Cancer”) AND (pesticide* OR herbicide* OR insecticide* OR fungicide* OR agrochemical*) AND (“Environmental Exposure” OR “Occupational Exposure” OR determinant*)
LILACS	((“neoplasias de cabeza y cuello” OR “cáncer oral” OR “cáncer de laringe” OR “cáncer de faringe”) AND (plaguicida* OR pesticida* OR herbicida* OR insecticida* OR agroquímico*) AND (“exposición ambiental” OR “exposición ocupacional” OR determinante*)
AGRICOLA	(“pesticides” OR “herbicides” OR “insecticides” OR “agrochemicals”) AND (“HNC” OR “oral cancer” OR “pharyngeal cancer” OR “laryngeal cancer”) AND (“occupational exposure” OR “environmental exposure” OR determinant*)

### Planned Outcomes and Variables to Be Extracted

In this systematic review, a set of critical and important outcomes will be extracted from the included studies to ensure a comprehensive analysis of determinants in patients with HNC associated with pesticides. Each variable will be clearly defined according to its analytical role in the review. Sociodemographic and occupational characteristics (eg, age, sex, occupation, place of residence, marital status, and ethnicity) will be treated as descriptive variables, whereas pesticide exposure time, type of pesticide, and exposure level will be considered primary determinants (exposures). Potential confounders such as smoking habits, alcohol consumption, and human papillomavirus infection status will be included where available to allow for adjusted interpretation of associations. Family medical history will also be considered as a relevant covariate.

Additionally, a set of contextual and methodological variables will be collected to support the interpretation and synthesis of findings. These include the database, author, and country of the study, and the participants’ access to health care as well as the objective of the study, the outcomes reported, and the key findings described in each publication.

### Data Collection and Selection Process

The selection process will follow a manual approach comprising title screening, abstract review, and full-text analysis. Data will be extracted into Microsoft Excel using the prespecified variables (eg, database, country, age, sex, occupation, exposure time, and pesticide type). Unavailable data will be requested from the study authors. An external researcher will serve as an impartial referee to resolve discrepancies.

Primary variables will include age, sex, occupation, duration of pesticide exposure, family history, and pesticide type. Structural stratifiers (eg, place of residence, occupation, ethnicity, and access to care) and intermediate determinants (eg, participant demographics and exposure specifics) will guide the narrative synthesis. Methodological and clinical heterogeneity will be assessed.

### Planned Presentation of Results

The results of the included studies will be systematically organized and synthesized in a comprehensive evidence table. This table will summarize key characteristics and findings extracted from each study, allowing for comparisons across study designs, populations, and exposure patterns. For each study, the following data will be reported to ensure a comprehensive and structured synthesis of the evidence: database and citation information, study design, participant demographics, geographic and occupational context, exposure details, ethnicity and lifestyle factors, health history and access to care, and study objectives and outcomes. These elements will facilitate consistent comparison across studies and support the identification of patterns, determinants, and gaps in the existing literature.

In addition, a narrative synthesis of the data will be conducted to complement the quantitative and qualitative findings. This synthesis will be structured according to the WHO conceptual framework of social determinants of health [10]: (1) structural determinants, which include broader sociopolitical and economic factors, such as income, education, occupation, social class, gender norms, and policies that influence health equity; and (2) intermediate determinants, which refer to the material circumstances, psychosocial factors, behavioral and biological factors, and access to health services that are shaped by structural determinants and directly impact individual health outcomes. This process will be performed by 2 authors and will have a peer-review process.

This thematic framework will guide the interpretation of the findings, helping to identify patterns and relationships between social determinants and the health outcomes reported in the included studies. The use of the WHO framework ensures a comprehensive and globally recognized approach to analyzing the complex social factors that influence health.

### Heterogeneity, Risk of Bias, and Certainty Assessment

The methodological heterogeneity of the included studies will be assessed based on the type of study design and the specific inclusion and exclusion criteria applied. Clinical heterogeneity will be examined by evaluating participant characteristics, such as age, sex, and comorbidities [11].

To assess the risk of bias in nonrandomized studies, we will use the ROBINS-I (Risk of Bias in Non-Randomized Studies of Interventions) tool, developed by the Cochrane Bias Methods Group [12]. ROBINS-I evaluates bias across 7 domains: confounding, participant selection, classification of interventions, deviations from intended interventions, missing data, measurement of outcomes, and selection of the reported result. Each domain is rated as low, moderate, severe, or critical risk of bias, or as having no information. This tool is particularly appropriate when synthesizing evidence from observational or quasi-experimental studies.

The certainty of evidence for each outcome will be assessed using the GRADE (Grading of Recommendations, Assessment, Development and Evaluations) approach [13].[13] GRADE considers 5 domains that may lower the certainty of the evidence: risk of bias, inconsistency, indirectness, imprecision, and publication bias. The quality of evidence will be categorized as high, moderate, low, or very low, providing a transparent and structured assessment of confidence in the effect estimates. This process will guide the strength and reliability of the conclusions drawn in the review.

### Ethical Considerations

This study did not require ethics committee review or approval because it is a systematic review based only on an analysis of previously published studies. No primary data were collected and no direct human participation took place. Therefore, ethical approval and informed consent were not applicable.

## Results

This systematic review was initiated in February 2025 after protocol registration. The literature search and review process is ongoing at the time of submission of this paper. Data extraction and quality assessment have not yet been completed. Final results of the review, including a synthesis of determinants associated with pesticide exposure in patients with HNC, are expected to be completed and submitted for publication in June 2026.

The findings of this systematic review will be presented through a structured synthesis of the included studies. A comprehensive evidence table will summarize essential study characteristics, such as database and citation information, study design, sample size, participant demographics (eg, age, sex, and ethnicity), geographic and occupational context, pesticide exposure type and duration, and cancer subtype diagnosed. Outcomes of interest, including critical and contextual variables, will be extracted and reported systematically.

Patterns in pesticide exposure and their potential determinants with HNC will be analyzed. The review will seek to identify common determinants among affected populations, such as occupational exposure, geographic distribution, and sociodemographic characteristics. Stratified analyses will be conducted, when possible, based on key variables such as pesticide type or exposure duration. Discrepancies between studies and data gaps will be noted, and findings will be synthesized narratively and, if feasible, quantitatively through meta-analysis.

**Figure 1.**
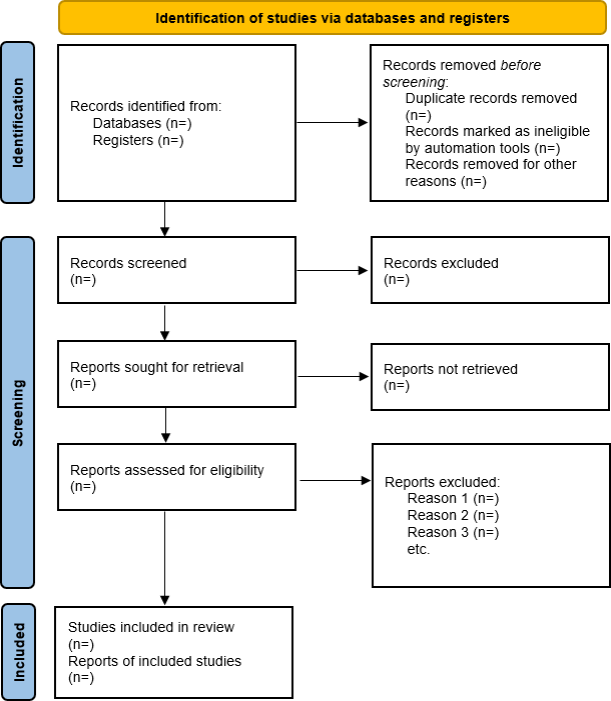
PRISMA-P (Preferred Reporting Items for Systematic Review and Meta-Analysis Protocols) selection flowchart.

A flow diagram following PRISMA-P guidelines will be used to report the selection process, including the number of records identified, screened, included, and excluded, along with reasons for exclusion (Figure 1). Methodological and clinical heterogeneity will be described, and the risk of bias and certainty of evidence will be reported for each study included. The results are intended to be published in December 2026.

## Discussion

### Anticipated Findings

The systematic review will provide a detailed synthesis of the current scientific evidence regarding the association between pesticide exposure and HNC. It will examine the role of environmental determinants—particularly those linked to agricultural or occupational settings—in the development of malignancies of the oral cavity, pharynx, and larynx. Through an in-depth evaluation of the literature, the review will explore whether consistent patterns emerge in terms of risk factors, populations affected, and exposure characteristics.

Previous studies have suggested that prolonged exposure to organophosphates, carbamates, and other agricultural pesticides may increase the risk of developing head and neck malignancies. For instance, epidemiological investigations conducted in agricultural communities have reported higher incidence rates of laryngeal and oral cancers among workers exposed to these compounds compared to nonexposed populations. Similarly, systematic reviews and meta-analyses have identified a potential dose-response relationship between cumulative pesticide exposure and cancer risk, although the strength of this association remains inconsistent across studies.

Findings will be interpreted considering the methodological quality and clinical diversity of the included studies. Particular attention will be given to how structural determinants—such as rural residency, type of employment, or limited access to health care—may modulate risk in vulnerable populations. The review will also discuss the potential influence of behavioral and intermediate factors such as tobacco or alcohol use, which may interact with pesticide exposure and contribute to cancer risk.

The discussion will critically assess limitations of the available evidence, including possible publication bias, insufficient exposure assessment methods, or lack of adjustment for confounders. Additional limitations may include heterogeneity in the definition and measurement of pesticide exposure across studies, retrospective study designs that limit causal inference, and underreporting of environmental or occupational data in low- and middle-income countries. Furthermore, the variability in diagnostic criteria for HNC subtypes could contribute to inconsistencies in observed associations. These limitations will make recommendations for future research, highlighting the need for more robust, longitudinal studies that integrate detailed environmental and occupational exposure data. Future investigations should also aim to standardize exposure metrics, incorporate biomonitoring approaches, and control for behavioral and genetic modifiers of susceptibility to better elucidate the causal pathways involved.

Ultimately, the results of this systematic review will contribute to a better understanding of the environmental determinants of HNC, informing the development of targeted public health strategies and occupational safety policies. The synthesis of current knowledge will also support clinicians and researchers in identifying at-risk populations and formulating hypotheses for future investigations.

### Conclusions

This systematic review will aim to fill a critical gap in the scientific literature by synthesizing existing evidence on determinants in patients with HNC associated with pesticides. Through a rigorous and transparent methodology, the review will identify key environmental and demographic determinants that may contribute to cancer risk in exposed populations. The findings are expected to provide a clearer understanding of how pesticide exposure, particularly in occupational and rural settings, may influence the development of malignancies in the oral cavity, pharynx, and larynx. The review will also highlight the importance of integrating environmental health perspectives into cancer prevention strategies and public health policy.

Ultimately, this work will support the identification of high-risk groups, inform regulatory decision-making regarding pesticide use, and guide future research aimed at mitigating environmental carcinogenic exposures. By establishing a structured and replicable approach, the review will contribute to the scientific community’s ability to address complex interactions between environmental hazards and cancer epidemiology.

## Supplementary material

10.2196/81312 Checklist 1 PRISMA-P checklist.

## Abbreviations

GRADE: Grading of Recommendations, Assessment, Development, and Evaluations
HNC: head and neck cancer
PECOS: population, exposure, comparator, outcome, and study
PRISMA-P: Preferred Reporting Items for Systematic Review and Meta-Analysis Protocols
ROBINS-I: Risk of Bias in Non-Randomized Studies of Interventions I
WHO: World Health Organization
